# Health inequities in functional limitation among Mexican older adults: An intersectional approach

**DOI:** 10.1371/journal.pone.0325211

**Published:** 2025-08-05

**Authors:** Héctor García-Hernández, Guillermo Salinas-Escudero, Hortensia Reyes-Morales, María Fernanda Carrillo-Vega

**Affiliations:** 1 Health Research Division, National Institute of Geriatrics, Mexico City, Mexico; 2 Center for Economic and Social Studies in Health, Hospital Infantil de México Federico Gómez, Mexico City, Mexico; 3 Center for Health Systems Research, National Institute of Public Health, Morelos, Mexico; 4 Researcher, National Institute of Geriatrics, Mexico City, Mexico; Universidad Santiago de Cali, COLOMBIA

## Abstract

Functional limitation represents a major health concern among older adults, with its incidence increased based on personal characteristics such as being a woman, having minor levels of education, and lower socioeconomic status, leading to health inequities. Addressing these inequities requires comprehensive frameworks like intersectionality to provide a broader perspective. This study analyzes health inequities in functional limitation among Mexican older adults using data from the 2021 round of the Mexican Health and Aging Study (MHAS) within an intersectional framework. The Multilevel Analysis of Individual Heterogeneity and Discriminatory Accuracy (MAIHDA) technique, recognized as the gold standard in quantitative intersectionality research, was employed. Six variables were assessed: age, sex, education, social engagement, economic status, and access to health services. The results indicate that age, social engagement, and economic status were the main variables that explain functional limitation. Enhancing social engagement emerges as a practical short-term strategy to improve functionality and reduce inequities. Contrary to prior evidence, sex was not directly associated with functional limitation. Therefore, higher rates of functionality loss previously reported in the literature may not simply be linked to being a woman but rather to the societal implications of being a woman in contemporary contexts. Similarly, access to health services did not show a significant relationship with functional limitation despite the health system being a critical intermediate social determinant of health with the potential to address inequities. This research underscores the importance of intersectionality in understanding inequality, offering a nuanced perspective on overlapping systems of oppression and privilege to address disparities in Mexican older adults.

## Introduction

Life expectancy has continuously increased since the Industrial Revolution due to improved life conditions, public health interventions, and scientific findings [1,2]. This trend has led to a steadily aging global population. In Mexico, the Law of the Rights of Older Adults (*Ley de los Derechos de las Personas Adultas Mayores*) defines an older adult as an individual aged 60 years or older [3]. This population grew from 6% of the total population in 1990 to 12% in 2020 [4,5]. The rise in the number of older adults has created an epidemiological scenario characterized by chronic diseases [6] and a reduction of functionality [7]. A research study reported an increase in disability among Mexican older adults, from 13.1% to 15.4% between 2012 and 2015 [8]. In 2020, 21% of older adults in Mexico were living with a disability [9].

The loss of functionality varies based on economic, social, ethnic, or cultural factors, thus leading to inequity [10,11]. It’s well known that lower socioeconomic status [12], having minor levels of education [13], or being a woman [8,13] increases the probability of having a disability in older adults. However, the processes or mechanisms from which these differences exist are not totally clear [14]. Certain limitations of classical epidemiological research overlook the complete understanding of health inequities—unjust disparities in health outcomes rooted in non-biological factors. It often assumes that the characteristics involved in the genesis of inequality are independent. However, these attributes must be fully studied since they are closely interrelated, and this interrelation significantly shapes inequality [15,16]. Analyzing one attribute at a time leads to identifying risk profiles at the individual level, resulting in interventions focused on the person, leaving behind the contextual factors that significantly impact personal health. Such studies also rely on group averages for comparisons, which offer limited predictive power in distinguishing cases and non-cases. These comparisons are typically made between the most and least advantaged individuals or groups, overlooking the differences within the middle groups, often excluding them from the public health agenda [17,18]. Consequently, inequity must be analyzed using more comprehensive frameworks that thoroughly explain how individual, or group attributes define and maintain health inequities.

Intersectionality has been proposed to address these limitations. As a theoretical framework, it posits that individuals belong to multiple social categories based on characteristics, such as sex, religion, race, education, income, etc. Each of these characteristics is linked to power relations, which are expressed through societal advantages or disadvantages (e.g., racism, sexism, xenophobia, homophobia). These attributes are interrelated and shape an individual’s “social location” within the social structure. This location determines differential access to resources and opportunities through complex, interconnected systems of privilege and oppression at the structural level. As an analytical framework, intersectionality argues that a comprehensive understanding of inequity can only be achieved by examining the intersections of these attributes at both individual and group levels [19–23]. Therefore, applying intersectionality in epidemiology allows for a more accurate capture of the structural mechanisms that produce health inequities, and facilitates an understanding of how social systems shape the inequities experienced by each individual [24]. Since its conceptualization in 1989 [25], intersectionality has been predominantly explored through qualitative studies, while quantitative intersectional analyses have only recently emerged and remain in early stages of use. Additionally, key variables often analyzed include sex, ethnicity, and socioeconomic factors, though inequities in disability have been poorly explored [26].

By using the intersectional quantitative approach, it has been possible to gain a deeper understanding of inequity. For example, in the United States, opioid addiction has traditionally been associated with white, middle-class individuals. However, intersectional analysis has revealed that other ethnic groups—such as African American and Latino individuals with higher incomes—also experience significant levels of opioid addiction [27]. These groups have often been overlooked in public policies aimed at addressing this problem. Additionally, intersectionality has provided insights into the “Mexican paradox” which refers to the phenomenon where Latino-Mexican individuals in the U.S. exhibit better health outcomes for certain conditions compared to white Americans. It has been suggested that the type of employment among Latino-Mexican individuals contributes significantly to their lower levels of disability [28].

Consequently, the intersectional framework can be used to gain a comprehensive understanding of the complexity of health inequities [15]. Applying an intersectional analysis is essential when studying functionality among Mexican older adults, as they face significant inequities [29] and a growing prevalence of functionality loss [8,9]. Therefore, this research aims to apply the intersectional quantitative approach to analyze health inequities in functional limitation among older adults.

## Materials and methods

### Study population

This is a secondary analysis of the 2021 round from the Mexican Health and Aging Study (MHAS), a nationally representative cohort of individuals 50 or older in Mexico. Information on the survey can be found on its official webpage (https://www.mhasweb.org). The 2021 sample consists of 17,538 interviews, from which 15,739 were alive, and 1,799 were Next-of-Kin. We included 15,257 observations of living adults of 50 years or more. From those observations, 15,248 answered the questions related to functionality.

### Variables

The outcome variable was functional limitation, which was defined if individuals respond to having problems performing any basic activities of daily living (BADLs) (eating, dressing, getting in or out of bed, bathing, walking, and using the toilet) and/or any instrumental activities of daily living (IADLs) (shopping, preparing food, managing money, and taking medications) [30–32].

We considered the Social Determinants of Health framework [33] to guide the selection of predictors, aiming to include structural and intermediary determinants relevant to functional decline and health inequities among older adults. These included age (50–59, 60–69, 70–79, and 80 or more years), sex (male and female), education (with or without at least one year of formal education), social engagement (if the person has a job or if he/she performs a voluntary, religiosity or other type activity for at least one hour per week during the last two years [34]), economic status (using the question “How do you consider your economic situation?” [35] with responses: excellent, very good, good, fair, and bad. These were categorized as good (including excellent, very good, and good), fair, and bad, and access to health services (defined if the person reported affiliation to any Mexican health system institution).

### Statistical analysis

We employed the Multilevel Analysis of Individual Heterogeneity and Discriminatory Accuracy (MAIHDA). MAIHDA is widely recognized as the gold standard for studying intersectionality through quantitative methods. It provides a comprehensive understanding of inequities by accounting for all possible interactions between variables within a multilevel regression analysis, considering the individual level (level 1) and the strata level (level 2) in which they are nested [36]. Strata level is defined by the intersection of selected variables delineating specific groups—“strata”— [17,18].

The first step is to assign each individual to specific strata, assuming that individuals within each strata share a similar “social position”. Constructing the strata involves generating all possible combinations of the selected variables [36]. This process involves considering each variable’s categories and combining them systematically to form distinct groups. In our study, this resulted in 192 unique strata (4x2x2x2x3x2). It’s recommended to exclude strata with five or fewer observations, as estimations from such small groups tend to have lower accuracy [37]. We analyzed strata characteristics by describing all strata with their probability of having functional limitations.

Then, different logistic regression models with the binary outcome—the presence or absence of functional limitation—were calculated: null or simple (Model 1), partially adjusted (Models 2A-2F), and main effect models (Model 3). Odds ratios (OR) were estimated with 95% confidence interval (95%CI) in each one. The null model is unadjusted; it specifies how the mean health outcome varies within and between strata [20,36]. Models 2A-2F correspond to each of the variables included in the analysis and were used to assess the extent to which each variable contributes to the between-strata variance, thereby highlighting the importance of each variable in functional limitation. Subsequently, Model 3 was calculated by introducing all six variables.

With these models, we calculate certain statistics to analyze intersectionality: the variance partition coefficient (VPC), the Area Under the Receiver Operating Characteristic Curve (AUC), and the Between-Strata Variance (PCV). The first one represents the proportion of individual variation at the strata level and reflects the influence that the context in which a person lives has on the health outcome [20,36,37]. Conventionally, the VPC is expressed as 0–1% (no influence), > 1- ≤ 5% (small influence), > 5- ≤ 10% (considerable influence), > 10- ≤ 20% (moderate to high influence), > 20- ≤ 30% (strong influence), and >30% (highly significant influence) [37]. A higher VPC indicates greater similarity among individuals within each strata. The VPC in the null model reflects the explanatory power of the intersectional strata, including both additive and potential multiplicative effects of the variables defining the strata. Thus, in Model 3, VPC indicates the percentage of the total variance explained by multiplicative effects at the strata level after controlling for additive effects. Therefore it represents the strata-level variance attributable solely to multiplicative effects [20].

In the intersectional approach, the AUC represents the probability that a randomly selected person with a functional limitation belongs to a strata with a higher odd of functional limitation compared to a randomly selected person without a functional limitation. The AUC ranges from 0.5 to 1, where 1 indicates perfect discriminatory accuracy and 0.5 suggests that the strata have no discriminatory accuracy [36].

Finally, the PCV is useful for identifying which variables are most influential in creating inequity patterns in outcomes in Models 2A-2F and Model 3. A high PCV indicated that the new variable strongly differentiates outcomes between strata, while a low PCV suggests the variable has little impact on the observed disparities across strata. In Model 3, PCV indicates how much of the between-strata variance is explained by the additive main effects and multiplicative effects [38]. The PCV is expressed as a percentage. Consequently, 1 − PCVs represents the percentage of the strata-level variance that cannot be explained by the additive effects and is therefore attributable to multiplicative effects [20]. See S1 File for more information related to MAIHDA.

The study is part of the research project *DI-PI-001/2024*, which was approved by the Research, Ethics, and Biosafety Committees of the Instituto Nacional de Geriatría. All the calculations were made with STATA v.17.

## Results

**Fig 1:**
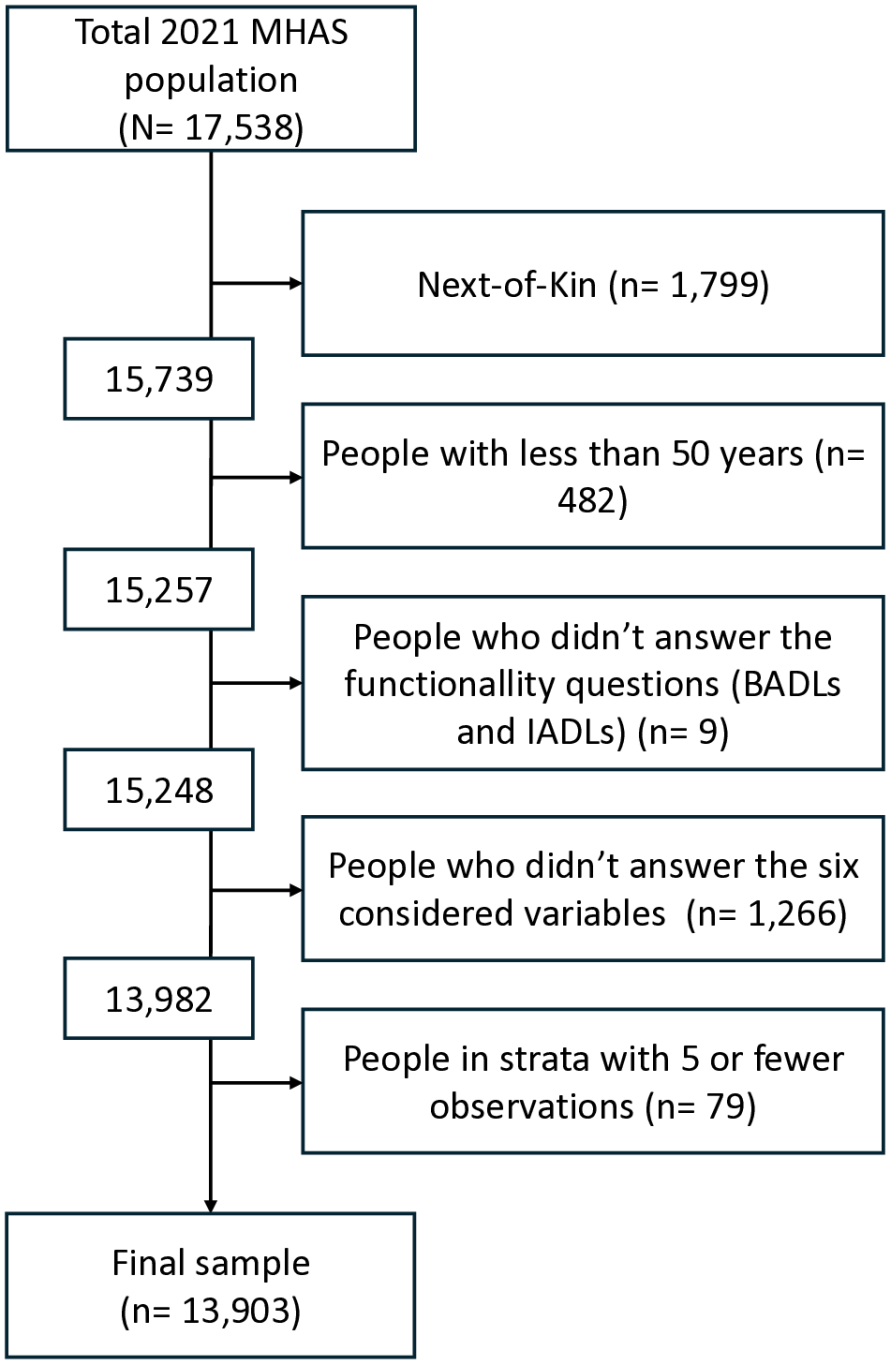
Sample selection of the 2021 MHAS survey. BADLs: Basic activities of daily living. IADLs: Instrumental activities of daily living.

Fig 1 shows the sample selection of the 2021 MHAS survey, from the 15,248 observations, 13,982 were considered to define the 192 strata. Table 1 shows the size distribution of these observations in the strata. The strata with 5 or fewer observations were eliminated. Consequently, from the 192 strata, 157 remain and represent 13,903 observations (Fig 1).

**Table 1:** Size distribution of the 192 constructed strata.

Size of strata	Number of strata
5 or less	35 (n = 79)
6 - 9	24 (n = 165)
10 - 19	36 (n = 489)
20 - 49	45 (n = 1,463)
50 - 99	17 (n = 1,198)
100 or more	35 (n = 10,588)

Table 2 reports the descriptive statistics of the final sample. From the 13,903 observations, 3,426 (24.64%) have functional limitations. Younger adults, males, with social engagement, and good socioeconomic status tended to have less functional limitations. Supporting Information 2 presents the strata ranking and its probability of functional limitations (S2 File). The consistent characteristics related to the outcome were age, social engagement, and economic status. The strata ranked 1 and 157 had a likelihood of functional limitation of 8.31% and 77.95%, respectively. The functional limitation probability of the most vulnerable strata was 9 times higher than that of the most protected strata.

**Table 2:** Descriptive analysis of the final sample.

	Total n = 13,903	
Variable	With functional limitation n = 3,426	Without functional limitation n = 10,477
Age		
50–59	37.21%	19.29%
60–69	33.26%	23.09%
70–79	23.50%	33.51%
80 or more	6.03%	24.11%
Sex		
Male	43.36%	38.59%
Female	56.64%	61.41%
At least one year of formal education		
With	90.97%	82.25%
Without	9.03%	17.75%
Social engagement		
With	49.28%	26.71%
Without	50.72%	73.29%
Economic status		
Good	35.29%	27.82%
Fair	59.49%	62.49%
Bad	5.22%	9.69%
Access to health services		
With	74.53%	74.99%
Without	25.47%	25.01%

Table 3 shows results from Model 1, Model 2A-F, and Model 3 from MAIHDA analysis. The VPC in Model 1 indicated that less than 20% of the total individual differences related to whether someone lost or did not have functional limitations were attributed to the strata to which they belonged. This value indicates a moderate to high contextual influence on the outcome. The PCV value from the variables included in Models 2A-F means that age, social engagement, and economic status were the most important factors in explaining variation in the probability of having functional limitations in older adults.

**Table 3:** Regression Models 1, 2A-2F, and 3.

	Model 1	Model 2A	Model 2B	Model 2C	Model 2D	Model 2E	Model 2F	Model 3
Variable	Null Model	Age	Sex	At least one year of formal education	Social engagement	Economic status	Access to health services	Multivariate Model
	OR (95%CI)	OR (95%CI)	OR (95%CI)	OR (95%CI)	OR (95%CI)	OR (95%CI)	OR (95%CI)	OR (95%CI)
Intercept	0.40^++^ (0.34-0.46)	0.21^++^ (0.17-0.26)	0.35^++^ (0.28-0.43)	0.34^++^ (0.28-0.41)	0.22^++^ (0.17-0.26)	0.28^++^ (0.22-0.35)	0.39^++^ (0.32-0.48)	0.09^++^ (0.08-0.11)
Age								
50–59 (reference)		–						–
60–69		1.24 (0.93-1.65)						1.19^+^ (1.02-1.39)
70–79		2.24^++^ (1.69-2.96)						2.17^++^ (1.86-2.55)
80 or more		5.55^++^ (4.09-7.52)						5.28^++^ (4.38-6.40)
Sex								
Male (reference)			–					–
Female			1.29 (0.95-1.73)					1.16^+^ (1.03-1.30)
At least one year of formal education								
With (reference)				–				–
Without				1.56^++^ (1.16-2.11)				1.37^++^ (1.19-1.57)
Social engagement								
With (reference)					–			–
Without					2.88^++^ (2.23-3.71)			1.75^++^ (1.54-1.99)
Economic status								
Good (reference)						–		–
Fair						1.44^+^ (1.04-2.00)		1.37^++^ (1.21-1.55)
Bad						2.44^++^ (1.66-3.56)		2.46^++^ (2.05-2.96)
Access to health services								
With (reference)							–	–
Without							1.05 (0.78-1.42)	1.02 (0.91-1.15)
Summary statistics								
Variance Partition Coefficient (VPC)	17.35%	6.64%	17.11%	15.94%	11.55%	15.46%	17.36%	0.68%
Proportional Change in Variance (PCV)	–	66.15%	1.70%	9.70%	37.78%	12.90%	0.00%	96.7%
Area Under Receiver Operating Characteristic Curve (AUC)	0.72	0.71	0.72	0.72	0.72	0.72	0.72	0.71

**Note:** Values represent odds ratios. Confidence intervals (CI) at 95% are found at the end of every cell.

+ p < 0.05.

++ p < 0.01.

After adjusting for the six variables, the VPC and PCV were 0.68% and 96.7%, respectively. These findings suggest that the differences in functional limitations across strata are largely explained by the additive effects of the variables—that is, each factor independently contributes to the observed inequities. However, the remaining unexplained variance indicates that not all disparities can be attributed to additive effects alone. The interaction between characteristics creates unique configurations of disadvantage that go beyond the isolated influence of each variable. Thus, the results from Model 3 underscore the limitations of a purely additive approach and highlight the relevance of intersectional in understanding inequities in functional limitations.

As described in the methodology, the key statistical measures used to interpret the intersectional model are the VPC, PCV, and AUC; the OR values must be interpreted considering these measures. Accordingly, Model 3 presents the ORs for the main effects. The strata that contain more older adults from 70−79 (OR:2.17; 95%CI:1.86–2.55) and 80 and more years (OR:5.28; 95%CI:4.38–6.40), the ones with more people that report bad economic status (OR:2.46; 95%CI:2.05–2.96), less social engagement (OR:1.75; 95%CI:1.54–1.99), less education (OR:1.37; 95%CI:1.19–1.57) and with more women (OR:1.16; 95%CI:1.03–1.30) increase their probability of having functional limitations. Finally, all the AUC values show an acceptable discriminatory accuracy [39]. This indicates that a randomly chosen individual experiencing functional limitations is likelier to belong to a stratum with a higher predicted probability of such outcome than an individual without functional limitations.

## Discussion

To our knowledge, this is the first research studying inequity in functional limitations in older Mexican adults using the intersectional framework and incorporating less frequently analyzed variables like health system and social engagement. Despite our findings aligning with classical epidemiological research, we provide key insights that enhance the understanding of disparities in functional limitations by demonstrating how the interaction between multiple variables contributes to the definition of inequities. So, this approach offers an integral, rather than partial, perspective. The main findings were: 1) age, social engagement, and economic status are the most important variables explaining the functional limitations in strata. 2) Contrary to prior evidence, sex didn’t have a relation with the outcome. 3) Access to health services didn’t relate to the outcome and 4) not all inequity is explained by additive effects. Therefore, the social context in which the individual lives plays a role in the differences in functional limitations.

Regarding the first finding, the VPC value in Model 1 indicates that the stratum where individuals are nested accounts for a significant variation in functional limitations, highlighting the contextual influence. This suggests the role of structural social determinants of health (SDH) in explaining disparities. Meanwhile, PCV values from Model 2 A-F reveal the contribution of specific variables to the between-intersection variance. Through the intersectional framework, we identified how these variables interact to create vulnerability patterns, highlighting their combined influence on functional limitations rather than considering them in isolation. As expected and reported previously, age is the main variable that reduces functionality [40–43], followed by social engagement, and economic status.

Functionality reduction is strongly related to age because of a progressive decline through time that can be slowed down with physical activity and healthy diets [44]. Thus, the link between age and functional limitation may reflect this process in our analysis. While age may have limited implications for functional inequities, other outcomes—such as abuse, mental health issues, cognitive decline, and poor quality of life—are significantly affected by the negative social perception of aging—ageism— [45]. Therefore, it’s important to include age as an intersection to completely understand the impact of ageism in shaping health differences [24,45–47].

As said before, SDHs are important in producing and maintaining inequities. Since 1790, poverty has been recognized as a root cause of diseases [48]. Subsequent scientific literature has supported this statement, indicating that lower economic status is associated with increased frequency of almost all diseases [49]. Regarding disability, it’s globally acknowledged that good economic status is linked to a lower likelihood of disability. Studies in Brazil [40], Spain [41], and Mexico [13] show a gradient where higher income is associated with a lower probability of disability. Education is another SDH closely related to inequity; globally, higher educational attainment is linked to lower disability rates [13,40,42,43,50–52]. Despite this, our findings show a PCV value for education of 9.70%, which means that a small percentage of differences between strata accounted for this variable. This may reflect the structural hierarchy of SDH [33]. Some authors argued that the only effective intervention to reduce health inequities is addressing the gaps in economic status, as social class significantly shapes inequity [53]. For example, a wealthy Black woman faces lower inequities than a poor Black woman, so this mix of privilege and oppression attributes may have a hierarchical influence on health outcomes [17].

However, economic status and education are difficult to modify to improve functionality as they require systemic, long-term efforts rather than direct interventions. We identified social engagement as a key modifiable factor, with a PCV value of 37.78%, which significantly explains disparities between strata. Social engagement is active participation in formal and non-formal community organizations. It reflects the integration of individuals in the diverse dynamics of society that imply interactions with others [54]. Research highlights its strong association with lower mortality [55], improved mental health [54], reduced chronic morbidity [56], and maintaining functionality. In the Netherlands, older adults with greater social networks and participation in activities had a lower disability risk compared to less engaged peers [50]. This is also true in Brazil [43], the United States [57], and Japan [58,59]; the last one shows a positive gradient between increased social activities performed and a lower probability of having functional limitations [60,61]. Additionally, in Taiwan and Sweden, this protective relationship demonstrated a long-term effect [52], and a positive annual improvement in physical function [62], respectively. Consequently, social engagement is the main variable that could be promptly intervened to maintain functionality.

The mechanism through which functionality is preserved via social participation is the active realization of BADLs and IADLs. Their consistent use strengthens musculoskeletal and neural systems, helping to prevent age-related physical decline. Additionally, social networks offer greater access to support and foster a sense of belonging, further contributing to maintaining functionality [57,61].

Social engagement has been extensively studied and promoted in high-income countries, where various initiatives encourage older adults to participate in cultural, sports, religious, and recreational activities. In contrast, Latin America has limited programs promoting older adults’ social participation and lacks sufficient research on the benefits of such activities [54]. Furthermore, in Latin America, the primary social network for older adults is the family, which provides crucial financial, healthcare, and social support. In Mexico, intergenerational households play an important role in socialization for older people, principally provided by the spouse and children [63]. This situation highlights the absence of programs for long-term care in Mexico, which places the burden of care on families, leading to increased costs and caregiver strain [64]. Additionally, this issue requires urgent attention, as demographic changes—such as smaller household sizes, the decline of nuclear families, and the rise in single-person households—further complicate socialization for older adults [63].

Although the Mexican government has promoted social initiatives to enhance the social participation of older adults, such as the *PILARES para el Bienestar* policy [65] and the National Institute for Older Adults (*Instituto Nacional de las Personas Adultas Mayores, INAPAM*) [66], there is a need to improve the availability and accessibility of these activities at the national level. This can be achieved by raising awareness about them, ensuring their geographical proximity, and providing adequate transportation options. This implies adapting urban infrastructure, including wider sidewalks, ramps, smooth pavements, and the installation of elevators. Additionally, these activities should be financially accessible and designed with an intergenerational approach [67].

Concerning the second finding, by analyzing the intersectionality statistics—VPC and PCV—we found that sex didn’t have a significant relationship with functional limitations, as previously reported. There’s evidence that demonstrated a substantial association between disability and being a woman in European countries [41,42], Brazil [43], India [51], the United States [68], Canada [69], and Mexico [8,13]. Moreover, more complex indicators, like disability-free life expectancy (DFLE), show that globally, women live longer but with a higher disability. This has been known as the male-female health-survival paradox that is explained by women’s protective biological factors—such as female hormones—, lower exposure to risk factors —like violent behaviors—, healthier lifestyles —including reduced smoking and alcohol consumption—, more use of healthcare systems compared to men, and a greater ability to communicate health problems to physicians [40,68,70–75]. However, in our results, the PVC value for sex was 1.70% which reflects that this variable has little impact on the disparities across strata.

Using intersectionality, our results suggest that having functional limitations is not simply linked to *being* a woman but to what it *means* to be a woman. In contemporary societies, older women are often stereotyped as less competent, more dependent, submissive, and less intellectually capable than men, which leads to a more complex discrimination process because of the intersection of sexism with other types of discrimination [46]. For example, older men have more socialization activities than older women [45], face less discrimination at work [61], and have a better socioeconomic status [51]. This perception leads to the undervaluation of older women’s significant contributions, including their roles in economic productivity, the transmission of knowledge and experiences, intergenerational caregiving, and providing essential family support. However, it must be acknowledged that multiple social determinants, such as employment status, pension, and health status, were not considered in this analysis, yet they may impact the differences in functionality between sexes.

Another significant observation is that health system access showed no relationship with functional limitations. Health systems are often regarded as intermediate SDH with the potential to reduce inequities [33]. For instance, in Brazil, affiliation with the health system lowers the likelihood of disability in older adults [40]. Our analysis contrasts with this finding. There may be organizational and contextual issues within the Mexican health system that hinder the goal of restoring functionality among older adults. For instance, despite the high percentage of health system affiliation among older adults in Mexico, healthcare services are underutilized due to structural challenges such as overcrowding, long waiting times, shortages of resources in the public sector [76], and ageism among healthcare professionals [24,46,77]. This finding is deeply concerning because the health system must provide adequate medical attention to improve the health status of older people. However, this result requires a more in-depth analysis due to the segmentation of the Mexican health system based on social security status [78]. This division has generated different health outcomes among the population, driven by variations in the quality of services and the availability of resources [79,80]. Moreover, recent transformations within the system, including the centralization of health services for the population without social security and modifications in the structure of health institutions providing health services, have led to significant changes in health system affiliation rates and access to healthcare services [78]. Therefore, a more precise classification of this variable is recommended.

Although our findings are consistent with classical epidemiological approaches, they also reveal that not all inequities can be explained solely by additive effects. This confirms that analyzing inequities without an intersectional perspective may oversimplify their complexity by focusing only on individual characteristics, overlooking the interconnected nature of social determinants [15,16].

Finally, we identified vulnerable groups with higher risk of health inequity that may be targeted for specific interventions. Being in the stratum defined as “*older woman, with no education, low economic status, limited social engagement, and no access to the health system*” confers more than nine times the probability of having functional limitations compared to “*younger man, with education, good economic status, social engagement, and health system access*” (S2 File). Intersectionality enables us to recognize the most and least advantaged groups and other at-risk populations that could benefit from tailored interventions. This is relevant as policy interventions that lack an intersectional perspective tend to intervene solely in the most disadvantaged groups and overlook others who might also be vulnerable. This can lead to “threshold effects”, where individuals just above the intervention threshold are excluded [15,16,21]. For this reason, intersectionality has been compared to precision medicine [18], as it allows for more precise identification of groups that could benefit from specific interventions [22]. As shown in Supporting Information 2, the groups requiring intervention are predominantly composed of women with no social engagement and fair or poor socioeconomic status.

This study presents several strengths. It’s the first to explore inequities among Mexican older adults using an intersectional framework. It also benefits from a representative sample and employs the gold standard for quantitative intersectional analysis. Furthermore, the study incorporates less frequently analyzed intersecting variables like health system and social engagement. However, certain limitations must be acknowledged. First, the outcome measure does not directly reflect disability. Further, the selection of variables was limited by the sample size [36], which may have prevented the inclusion of relevant factors associated with the outcome. Analyzing other variables could offer a more understanding of the complex interactions among determinants that drive health inequities. However, as the number of variables used to define strata increases, so does the number of resulting strata, which can lead to insufficient observations within some of them, thereby restricting the feasibility of applying MAIHDA. To overcome these limitations, fulfill the methodological requirements necessary for the robust application of MAIHDA, and incorporate a broader range of variables—such as ethnicity, migration status, lifestyle behaviors, and chronic health conditions— larger and more comprehensive datasets are needed. Also, longitudinal studies could also provide a clearer understanding of the progression from preserved functionality to functionality loss, offering insights into the development and persistence of inequities [14]. Although MHAS is a longitudinal study, the sample size was insufficient to perform MAIHDA longitudinally. Expanding the dataset would allow a deeper examination of the temporal and structural mechanisms driving disparities. A final limitation is that this information may not be generalizable to populations outside Mexico, as the interactions of these predictors could vary in other countries. Therefore, the results should be interpreted carefully when applied to other populations.

## Conclusion

Adopting an intersectional approach allows for a more comprehensive understanding of health inequities by recognizing that individual characteristics are interconnected and shaped by context. This perspective moves beyond individual risk profiles and helps to explain the broader range of health disparities across populations. Our findings reveal that different groups experience varying functional limitations, enabling more targeted and effective interventions. Moreover, we demonstrate that enhancing social engagement represents a short-term strategy to improve functionality and promptly reduce inequities. Importantly, our study highlights that higher probabilities of disability are not inherently linked to *being* a woman but are shaped by societal perceptions and stereotypes about what it *means* to be a woman. Consequently, scientific findings on social determinants should be interpreted within the context of power dynamics and privilege. Regarding the health system, we recommend improving access for this age group and encouraging further research into its role in restoring and maintaining functionality. This will enable the provision of adequate medical attention and contribute to improving health outcomes of older populations.

## Supporting information

S1 File MAIHDA statistical description. (DOCX)

S2 File Strata ranking. (DOCX)

## References

[1] Bongaarts J. Human population growth and the demographic transition. Philos Trans R Soc Lond B Biol Sci. 2009;364(1532):2985–90. doi: 10.1098/rstb.2009.0137 19770150 PMC2781829

[2] Mackenbach JP. The contribution of medical care to mortality decline: McKeown revisited. J Clin Epidemiol. 1996;49(11):1207–13. doi: 10.1016/s0895-4356(96)00200-4 8892485

[3] Gobierno de México. Ley de los Derechos de las Personas Adultas Mayores [Internet]. 2002. Available from: https://www.diputados.gob.mx/LeyesBiblio/pdf/LDPAM.pdf

[4] Muñoz M, Muñoz V, Gutiérrez MT. La situación demográfica de México. 1ra edition. México: Consejo Nacional de Población; 2015.

[5] Instituto Nacional de Estadística y Geografía. Censo de Población y Vivienda 2020 [Internet]. Instituto Nacional de Estadística y Geografía. s.f. Available from: https://www.inegi.org.mx/programas/ccpv/2020/

[6] Parra-Rodríguez L, González-Meljem JM, Gómez-Dantés H, Gutiérrez-Robledo LM, López-Ortega M, García-Peña C, et al. The Burden of Disease in Mexican Older Adults: Premature Mortality Challenging a Limited-Resource Health System. J Aging Health. 2020;32(7–8):543–53. doi: 10.1177/0898264319836514 30913945

[7] Kánter I. Las personas mayores a través de los datos censales de 2020. 1ra ed. ed. México: Instituto Belisario Domínguez. 2021.

[8] Cabrero Castro JE, García-Peña C, Ramírez Aldana R. Transitions of disability, disability-free life expectancy and health insurance among adults aged 50 and older in Mexico: a multistate life table analysis. BMJ Open. 2021;11(8):e045261. doi: 10.1136/bmjopen-2020-045261 34353793 PMC8344280

[9] Instituto Nacional de Estadística y Geografía. Estadísticas a propósito del Día Internacional de las personas con discapacidad [Internet]. Instituto Nacional de Estadística y Geografía. 2021. Available from: https://www.inegi.org.mx/contenidos/saladeprensa/aproposito/2021/EAP_PersDiscap21.pdf

[10] Smith MD, Wesselbaum D. Global evidence of inequality in well-being among older adults. J Am Geriatr Soc. 2024;72(3):842–9. doi: 10.1111/jgs.18694 38038402

[11] Whitehead M, Dahlgren G. Conceptos y principios de la lucha contra las desigualdades sociales en salud. 1ra ed. ed. United Kingdom: WHO Regional Office for Europe; 2006.

[12] Barrantes M, García EJ, Gutiérrez LM, Miguel A. Dependencia funcional y enfermedades crónicas en ancianos mexicanos. Salud pública de México. 2007;49. 10.1590/s0036-36342007001000004 17724518

[13] Díaz C, De La Vega S, Wong R. Transitions in activities of daily living in Mexico, 2001-2012. Salud Publica Mex [Internet]. 8 de enero de 2015 [citado 29 de julio de 2024];57:54. Available from: http://www.saludpublica.mx/index.php/spm/article/view/7590 10.21149/spm.v57s1.7590 PMC4688039 26172235

[14] Pearlin LI, Schieman S, Fazio EM, Meersman SC. Stress, health, and the life course: some conceptual perspectives. J Health Soc Behav. 2005;46(2):205–19. doi: 10.1177/002214650504600206 16028458

[15] Barboza C, Sáenz JP, Fantin R, Gómez I, Rojas K. Theoretical Implications for the Analysis of Social Health Inequalities: A Discussion. Odovtos - Int J Dent Sc. 2019;15–25.

[16] Bowleg L. When Black + Lesbian + Woman ≠ Black Lesbian Woman: The Methodological Challenges of Qualitative and Quantitative Intersectionality Research. Sex Roles. 2008;59(5–6):312–25. doi: 10.1007/s11199-008-9400-z

[17] Evans CR, Williams DR, Onnela J-P, Subramanian SV. A multilevel approach to modeling health inequalities at the intersection of multiple social identities. Soc Sci Med. 2018;203:64–73. doi: 10.1016/j.socscimed.2017.11.011 29199054

[18] Merlo J. Multilevel analysis of individual heterogeneity and discriminatory accuracy (MAIHDA) within an intersectional framework. Soc Sci Med. 2018;203:74–80. doi: 10.1016/j.socscimed.2017.12.026 29305018

[19] Bersezio ME, Faúndez A, Quiroz S, Siclari P, Tarducci G. ¿Qué entendemos por interseccionalidad? 1ra edition. México: Consultora Inclusión y Equidad; 2020.

[20] Keller L, Lüdtke O, Preckel F, Brunner M. Educational Inequalities at the Intersection of Multiple Social Categories: An Introduction and Systematic Review of the Multilevel Analysis of Individual Heterogeneity and Discriminatory Accuracy (MAIHDA) Approach. Educ Psychol Rev. 2023;35(1). doi: 10.1007/s10648-023-09733-5

[21] Kelly C, Dansereau L, Sebring J, Aubrecht K, FitzGerald M, Lee Y, et al. Intersectionality, health equity, and EDI: What’s the difference for health researchers?. Int J Equity Health. 2022;21(1):182. doi: 10.1186/s12939-022-01795-1 36536361 PMC9764702

[22] Atewologun D. Intersectionality Theory and Practice. En: Oxford Research Encyclopedia of Business and Management [Internet]. Oxford University Press; 2018 [citado 8 de noviembre de 2024]. Available from: http://business.oxfordre.com/view/10.1093/acrefore/9780190224851.001.0001/acrefore-9780190224851-e-48

[23] Hancock A-M. Empirical Intersectionality: A Tale of Two Approaches. The Politics of Intersectionality. Springer International Publishing. 2019. p. 95–132. doi: 10.1007/978-3-319-98473-5_5

[24] Weßel M, Schweda M. Recognizing the Diverse Faces of Later Life: Old Age as a Category of Intersectional Analysis in Medical Ethics. J Med Philos. 2023;48(1):21–32. doi: 10.1093/jmp/jhac038 36519751

[25] Crenshaw K. Demarginalizing the Intersection of Race and Sex: A Black Feminist Critique of Antidiscrimination Doctrine, Feminist Theory and Antiracist Politics. University of Chicago Legal Forum. 1989;1(8).

[26] Bauer GR, Churchill SM, Mahendran M, Walwyn C, Lizotte D, Villa-Rueda AA. Intersectionality in quantitative research: A systematic review of its emergence and applications of theory and methods. SSM Popul Health. 2021;14:100798. doi: 10.1016/j.ssmph.2021.100798 33997247 PMC8095182

[27] Persmark A, Wemrell M, Evans CR, Subramanian SV, Leckie G, Merlo J. Intersectional inequalities and the U.S. opioid crisis: challenging dominant narratives and revealing heterogeneities. Critical Public Health. 2019;30(4):398–414. doi: 10.1080/09581596.2019.1626002

[28] Siordia C. Prevalence of Self-Care and Ambulatory Disability in Baby Boom and Generation-X Birth-Cohorts by Intersectional Markers of Social Stratification. Race Soc Probl. 2015;7(4):257–68. doi: 10.1007/s12552-015-9155-4

[29] Williams MM. Invisible, unequal, and forgotten: health disparities in the elderly. Notre Dame Journal of Law, Ethics & Public Policy. 2007;21(2):441–78.

[30] Katz S. Assessing self-maintenance: activities of daily living, mobility, and instrumental activities of daily living. J Am Geriatr Soc. 1983;31(12):721–7. doi: 10.1111/j.1532-5415.1983.tb03391.x 6418786

[31] Mino-León D, Giraldo-Rodríguez L, Rojas-Huerta A, Prado-Galbarro FJ, Reyes-Morales H. Multimorbidity, Functionality, Socioeconomic and Behavioral Conditions Linked with Mortality in a Cohort of Adults: A Latent Class Analysis. Arch Med Res. 2023;54(6):102869. doi: 10.1016/j.arcmed.2023.102869 37595496

[32] O’Young B, Gosney J, Ahn C. The Concept and Epidemiology of Disability. Phys Med Rehabil Clin N Am. 2019;30(4):697–707. doi: 10.1016/j.pmr.2019.07.012 31563163

[33] Orielle S, Irwin A. A conceptual framework for action on the social determinants of health. 1ra edition. Switzerland: World Health Organization; 2010.

[34] Howrey B, Avila JC, Downer B, Wong R. Social Engagement and Cognitive Function of Older Adults in Mexico and the United States: How Universal Is the Interdependence in Couples?. J Gerontol B Psychol Sci Soc Sci. 2021;76(Suppl 1):S41–50. doi: 10.1093/geronb/gbaa025 34101812 PMC8186856

[35] National Center for Education Statistics. Improving the Measurement of Socioeconomic Status for the National Assessment of Educational Progress [Internet]. 1st edition. United States: National Center for Education Statistics; 2012. Available from: https://eric.ed.gov/?id=ED542101

[36] Evans CR, Leckie G, Subramanian SV, Bell A, Merlo J. A tutorial for conducting intersectional multilevel analysis of individual heterogeneity and discriminatory accuracy (MAIHDA). SSM Popul Health. 2024;26:101664. doi: 10.1016/j.ssmph.2024.101664 38690117 PMC11059336

[37] He JW, Terry AL, Lizotte D, Bauer G, Ryan BL. Understanding intersectional inequality in access to primary care providers using multilevel analysis of individual heterogeneity and discriminatory accuracy. PLoS One. 2024;19(1):e0296657. doi: 10.1371/journal.pone.0296657 38241267 PMC10798491

[38] Axelsson Fisk S, Mulinari S, Wemrell M, Leckie G, Perez Vicente R, Merlo J. Chronic Obstructive Pulmonary Disease in Sweden: An intersectional multilevel analysis of individual heterogeneity and discriminatory accuracy. SSM Popul Health. 2018;4:334–46. doi: 10.1016/j.ssmph.2018.03.005 29854918 PMC5976844

[39] de Hond AAH, Steyerberg EW, van Calster B. Interpreting area under the receiver operating characteristic curve. Lancet Digit Health. 2022;4(12):e853–5. doi: 10.1016/S2589-7500(22)00188-1 36270955

[40] Alves LC, Leite I da C, Machado CJ. Factors associated with functional disability of elderly in Brazil: a multilevel analysis. Rev Saude Publica. 2010;44(3):468–78. doi: 10.1590/s0034-89102010005000009 20464262

[41] Abellán A, Rodríguez-Laso Á, Pujol R, Barrios L. A higher level of education amplifies the inverse association between income and disability in the Spanish elderly. Aging Clin Exp Res. 2015;27(6):903–9. doi: 10.1007/s40520-015-0345-0 25759168

[42] de Lima ALB, Espelt A, de Lima KC, Bosque-Prous M. Activity limitation in elderly people in the European context of gender inequality: a multilevel approach. Cien Saude Colet. 2018;23(9):2991–3000. doi: 10.1590/1413-81232018239.20662016 30281736

[43] de Lima ALB, Espelt A, Bosque-Prous M, Lima KC. Gender differences in disability among older adults in the context of social gender and income inequalities: 2013 Brazilian Health Survey. Rev Bras Epidemiol. 2020;23:e200002. doi: 10.1590/1980-549720200002 32130391

[44] Sadana R, Michel JP. Healthy Ageing: What Is It and How to Describe It? En: Michel JP, editor. Prevention of Chronic Diseases and Age-Related Disability [Internet]. Cham: Springer International Publishing; 2019 [citado 9 de julio de 2024]. p. 11–24. (Practical Issues in Geriatrics). Available from: http://link.springer.com/10.1007/978-3-319-96529-1_2

[45] Akerkar S. Gender and Older People [Internet]. 1st edition. United States: United Nation Department of Economic and Social Affairs; 2022. Available from: https://www.un.org/development/desa/ageing/wp-content/uploads/sites/24/2022/03/Gender-and-Older-People-Supriya-AKERKAR.pdf

[46] Merodio G, Martínez Ortiz de Zárate A, Zhu F, Morentin-Encina J. The Impact of Gendered Ageism and Related Intersectional Inequalities on the Health and Well-being of Older Women. RASP. 2024;12(2):146–65. doi: 10.17583/rasp.15017

[47] Walsh CA, Olson JL, Ploeg J, Lohfeld L, MacMillan HL. Elder abuse and oppression: voices of marginalized elders. J Elder Abuse Negl. 2011;23(1):17–42. doi: 10.1080/08946566.2011.534705 21253928

[48] Sigerist H. Johann Peter Frank: Un pionero de la medicina social. Salud Colectiva. 2006;2(3):269–79.

[49] Ortiz L, Pérez D, Tamez S. Desigualdad socioeconómica y salud en México. Revista Médica del Instituto Mexicano del Seguro Social. 2015;53(3):336–47. 25984619

[50] van Hees SGM, van den Borne BHP, Menting J, Sattoe JNT. Patterns of social participation among older adults with disabilities and the relationship with well-being: A latent class analysis. Arch Gerontol Geriatr. 2020;86:103933. doi: 10.1016/j.archger.2019.103933 31542633

[51] Anand A, Syamala T, Kanchan S k MI, Bhatt N. Understanding frailty, functional health and disability among older persons in India: A decomposition analysis of gender and place of resident. J Res Health Sci. 2020;20(3):e00484–e00484. 10.34172/jrhs.2020.20 PMC7585762 33169716

[52] Chang W-C, Lu F-P, Lan T-Y, Wu S-C. Multidimensional health-transition patterns among a middle-aged and older population. Geriatr Gerontol Int. 2013;13(3):571–9. doi: 10.1111/j.1447-0594.2012.00937.x 22985100

[53] Navarro V. What we mean by social determinants of health. Int J Health Serv. 2009;39(3):423–41. doi: 10.2190/HS.39.3.a 19771949

[54] Sepúlveda-Loyola W, Dos Santos Lopes R, Tricanico Maciel RP, Suziane Probst V. Participación social, un factor a considerar en la evaluación clínica del adulto mayor: una revisión narrativa. Rev Peru Med Exp Salud Publica. 2020;37(2):341–9. doi: 10.17843/rpmesp.2020.372.4518 32876227

[55] Holt-Lunstad J, Smith TB, Layton JB. Social relationships and mortality risk: a meta-analytic review. PLoS Med. 2010;7(7):e1000316. doi: 10.1371/journal.pmed.1000316 20668659 PMC2910600

[56] Singer L, Green M, Rowe F, Ben-Shlomo Y, Morrissey K. Social determinants of multimorbidity and multiple functional limitations among the ageing population of England, 2002–2015. SSM - Population Health. 2019;8:100413. 31194123 10.1016/j.ssmph.2019.100413 PMC6551564

[57] James BD, Boyle PA, Buchman AS, Bennett DA. Relation of late-life social activity with incident disability among community-dwelling older adults. J Gerontol A Biol Sci Med Sci. 2011;66(4):467–73. doi: 10.1093/gerona/glq231 21300745 PMC3055280

[58] Fujihara S, Tsuji T, Miyaguni Y, Aida J, Saito M, Koyama S, et al. Does Community-Level Social Capital Predict Decline in Instrumental Activities of Daily Living? A JAGES Prospective Cohort Study. Int J Environ Res Public Health. 2019;16(5):828. doi: 10.3390/ijerph16050828 30866468 PMC6427449

[59] Watanabe R, Tsuji T, Ide K, Saito M, Shinozaki T, Satake S, et al. Comparison of the Incidence of Functional Disability Correlated With Social Participation Among Older Adults in Japan. J Am Med Dir Assoc. 2024;25(6):104932. doi: 10.1016/j.jamda.2024.01.001 38336357

[60] Okura M, Ogita M, Yamamoto M, Nakai T, Numata T, Arai H. Community activities predict disability and mortality in community-dwelling older adults. Geriatr Gerontol Int. 2018;18(7):1114–24. doi: 10.1111/ggi.13315 29603568

[61] Tomioka K, Kurumatani N, Hosoi H. Association Between Social Participation and 3-Year Change in Instrumental Activities of Daily Living in Community-Dwelling Elderly Adults. J Am Geriatr Soc. 2017;65(1):107–13. doi: 10.1111/jgs.14447 27673582

[62] Saadeh M, Welmer A-K, Dekhtyar S, Fratiglioni L, Calderón-Larrañaga A. The Role of Psychological and Social Well-being on Physical Function Trajectories in Older Adults. J Gerontol A Biol Sci Med Sci. 2020;75(8):1579–85. doi: 10.1093/gerona/glaa114 32384140 PMC7357580

[63] Garay Villegas S, Montes de Oca Zavala V, Guillén J. Social Support and Social Networks Among the Elderly in Mexico. Population Ageing. 2014;7(2):143–59. doi: 10.1007/s12062-014-9099-2

[64] Attia RA, Abou FA. Caregiver burden from caring for impaired elderly: a cross-sectional study in rural Lower Egypt. IJPH. 2012;9(4). Available from: https://riviste.unimi.it/index.php/ijphjournal/article/view/22692

[65] Gobierno de la Ciudad de México. PILARES para el Bienestar [Internet]. s.f. Available from: https://pilares.cdmx.gob.mx

[66] Gobierno de México. Instituto Nacional de las Personas Adultas Mayores [Internet]. s.f. Available from: https://www.gob.mx/inapam

[67] Organización Mundial de la Salud. Ciudades globales amigables con los mayores: una guía. 1ra edition. Suiza: Organización Mundial de la Salud; 2007.

[68] Murtagh KN, Hubert HB. Gender differences in physical disability among an elderly cohort. Am J Public Health. 2004;94(8):1406–11. doi: 10.2105/ajph.94.8.1406 15284051 PMC1448463

[69] Raîche M, Hébert R, Dubois M-F, Gueye NR, Dubuc N. Covariates of Disability-Profile Transitions in Older People Living at Home. JBM. 2014;02(03):25–36. doi: 10.4236/jbm.2014.23005 22225577

[70] Chirinda W, Chen H. Comparative study of disability-free life expectancy across six low- and middle-income countries. Geriatr Gerontol Int. 2017;17(4):637–44. doi: 10.1111/ggi.12748 27197085

[71] Minicuci N, Noale M, León Díaz EM, Gómez León M, Andreotti A, Mutafova M. Disability-free life expectancy: a cross-national comparison among Bulgarian, Italian, and Latin American older population. J Aging Health. 2011;23(4):629–81. doi: 10.1177/0898264310390940 21220352

[72] Drumond Andrade FC, Guevara PE, Lebrão ML, de Oliveira Duarte YA, Santos JLF. Gender differences in life expectancy and disability-free life expectancy among older adults in São Paulo, Brazil. Womens Health Issues. 2011;21(1):64–70. doi: 10.1016/j.whi.2010.08.007 21185991

[73] Moreno X, Lera L, Albala C. Disability-free life expectancy and life expectancy in good self-rated health in Chile: Gender differences and compression of morbidity between 2009 and 2016. PLoS One. 2020;15(4):e0232445. doi: 10.1371/journal.pone.0232445 32353089 PMC7192428

[74] World Health Organization. Life expectancy and Healthy life expectancy [Internet]. Global Health Observatory data repository. s.f. Available from: https://apps.who.int/gho/data/node.main.688

[75] Wang S, Hu S, Wang P, Wu Y, Liu Z, Zheng H. Disability-Free Life Expectancy among People Over 60 Years Old by Sex, Urban and Rural Areas in Jiangxi Province, China. International Journal of Environmental Research and Public Health. 2021;18(9):4636. Available from: https://www.mdpi.com/1660-4601/18/9/4636 33925511 10.3390/ijerph18094636 PMC8123896

[76] García H, Pérez MU, Parra L, García C. La cascada de acceso al sistema público de salud en personas mayores mexicanas y factores asociados. GMM. 2024;160(3):15044. Available from: https://gacetamedicademexico.com/frame_esp.php?id=943 10.24875/GMM.24000074 39602615

[77] García C, Medina RH, Pérez MU, Vázquez J, Terán D, De La Torre A. Personas mayores olvidadas: deuda del sistema de salud. GMM. 2024;160(3):14266. Available from: https://gacetamedicademexico.com/frame_esp.php?id=941 10.24875/GMM.M24000894 39602618

[78] García H, Esquer D. Análisis comparativo de los sistemas de salud de México y Colombia. Población y Salud en Mesoamérica. 2024;21(2):17.

[79] García Hernández H, Dávila Cervántes CA. Análisis de la mortalidad evitable en México durante el periodo 1998-2019. PSM. 2022. Available from: https://revistas.ucr.ac.cr/index.php/psm/article/view/50116

[80] García H, García RE, Pérez MU, García C. Association between the changes in social- security continuity condition and mortality: MHAS 2001-2018 analysis. Salud Pública de México. 2023;65(5). 10.21149/14727 PMC10751061 38060919

