# Supplementary material for: Health inequities in functional limitation among Mexican older adults: An intersectional approach
Source: PLoS One. 2025 Aug 5;20(8):e0325211. doi: 10.1371/journal.pone.0325211 (PMC12324098; doi:10.1371/journal.pone.0325211)
Supplement: S1 File — (DOCX) [file pone.0325211.s001.docx]

**Supplementary Material 2. MAIHDA statistical description.**

The Multilevel Analysis of Individual Heterogeneity and Discriminatory Accuracy (MAIHDA) is the gold standard for studying intersectionality through quantitative methods. It can be used through frequentist maximum likelihood estimation (MLE) or a Bayesian approach. In this research, we use the first one. MAIHDA provides a comprehensive understanding of inequities by accounting for all possible interactions between variables within a multilevel regression analysis, considering level 1 (the individual level) and level 2 (the strata level) (1). Strata level is defined by the intersection of selected variables delineating specific groups (2,3). Also, MAIHDA offers a thorough analysis of variables by examining the statistical multiplicative effects of interactions, rather than their additive effects. The latter approach treats categories as independent advantages or disadvantages, overlooking their integrative effects (2).

In contrast to classical regression models with interaction terms, MAIHDA allows for more precise estimations even with small sample sizes within strata, as each strata becomes smaller with more variables analyzed. Additionally, these models are more parsimonious, as they increase the number of interactions geometrically rather than exponentially (4). Furthermore, MAIHDA decomposes the variance between and within intersectional strata to evaluate the heterogeneity of health outcomes at the individual and strata levels (3).

The first step is to assign each individual to a specific strata, assuming that individuals within each strata share a similar "social position" of oppression and privilege. The construction of strata involves creating all possible combinations of the selected variables (1). It’s recommended to exclude strata with 5 or fewer observations, as estimations from such small groups tend to have lower accuracy (5).

Then, different regression models are calculated: null or simple (Model 1), partially adjusted (Model 2), and main effect (Model 3). The null model is unadjusted, focusing on individuals nested within strata. This model explains how the total variance of the health outcome is attributed to the variance between intersectional strata and individual differences within those strata. Thus, the null model specifies how the mean of the health outcome varies within and between strata. The method for calculating regression models differs depending on the type of outcome. In this research, we used a binary outcome (presence or absence of functional loss), so odd ratios (OR) were estimated with 95% confidence interval (95%CI).

The null model for logistic regression represents the probability of being in that state (1,4). The model can be expressed through the following equations:

$$y_{ij}\sim Bernoulli(\pi_{j})$$

$$logit\left( \pi_{j} \right)=log\left( \frac{\pi_{j}}{1-\pi_{j}} \right)=\alpha_{o}+\mathcal{u}_{j}$$

$$\mathcal{u}_{j}\sim N(0,\sigma_{\mathcal{u}}^{2})$$

where $y_{ij}$​ denotes the presence of the health outcome for individuals $i$ in strata $j$, while $\pi_{j}$ is the probability of loss of functionality, assuming a Bernoulli distribution. The logit equation represents the logarithm of the odds of loss of functionality. $\alpha_{o}$​ shows the intercept of the model before adding any other factor and $\mathcal{u}_{j}$​ is the random effect term at the strata level. The $\mathcal{u}_{j}$ is assumed to be normally distributed. This term captures the variability between different strata that cannot be explained by the fixed effects of the model.

Then, the partially adjusted models are performed, each corresponding to one of the variables included in the analysis. These models are used to understand the extent to which each variable contributes to the between-strata variance, thereby distinguishing the importance of each variable in the outcome. Subsequently, Model 3 considers all variables. The following equation denotes the addition of the additive main effects to the fixed effects for Models 2 and 3 (1):

$$y_{ij}\sim Bernoulli(\pi_{j})$$

$$logit\left( \pi_{j} \right)=\alpha_{o}+\alpha_{1}x_{1j}+\alpha_{2}x_{2j}+\ldots+\mathcal{u}_{j}$$

$$\mathcal{u}_{j}\sim N(0,\sigma_{\mathcal{u}}^{2})$$

The different models provide key statistics that summarize the overall inequity in the whole sample. In the null model, the Variance Partition Coefficient (VPC), also known as the Intra-class Correlation Coefficient (ICC), is a global measure of intersectionality. It summarizes the outcome heterogeneity within and between strata without considering other factors (1,4,5). The VPC represents the proportion of individual variation in $y_{ij}$ at the strata level. Consequently, this value provides information about the General Contextual Effect (GCE), which reflects the influence that the context in which a person lives has on the health outcome. A higher VPC value indicates greater similarity among individuals within each strata. The VPC for logistic regressions was calculated using the following equation:

$$VPC=\frac{\sigma_{\mathcal{u}}^{2}}{\sigma_{\mathcal{u}}^{2}+\frac{\pi^{2}}{3}}$$

where $\sigma_{\mathcal{u}}^{2}$ represents the between-strata variance, and $\frac{\pi^{2}}{3}$​ denotes the variance of a standard logistic distribution. Conventionally, the VPC is expressed as a percentage by multiplying by 100. The VPC is classified as follows: 0%-1% (no relation, suggesting no difference attributable to the context), >1%-≤5% (poor variability, indicating a small contextual influence), >5%-≤10% (fair, suggesting a considerable influence of the context), >10%-≤20% (good, indicating a moderate to high contextual influence), >20%-≤30% (very good, which means a strong influence of the context in the outcome), and >30% (excellent, which shows a highly significant context influence in results). After adjusting the VPC with the variables introduced in Model 3, the adjusted VPC indicates the percentage of the total variance explained by multiplicative effects at the strata level after controlling for additive effects. Thus, the VPC in the null model reflects the explanatory power of the intersectional strata, including both additive and potential interaction effects of the variables defining the strata. While the adjusted VPC represents the strata-level variance attributable solely to interaction effects (4).

Additionally, the VPC can be used to calculate the Area Under the Receiver Operating Characteristic Curve (AUC), which is a measure of discriminatory accuracy and assesses how well the model distinguishes between individuals with and without the outcome. In the intersectional approach, the AUC represents the probability that a randomly selected person with the outcome belongs to a strata with a higher odd loss of having the outcome than a randomly selected person without it. The AUC ranges from 0.5 to 1, where 1 indicates perfect discriminatory accuracy and 0.5 suggests that the strata have no discriminatory accuracy (1).

Furthermore, in Models 2 and 3, the Proportional Change in the Between-Strata Variance (PCV) can be calculated to determine the proportion of variance in the base model (Model 1) that is reduced when a new variable is added to the subsequent models (Models 2 and 3). It shows how much of a specific variable contributes to explaining differences across strata. A high PCV indicated that the new variable strongly differentiates outcomes between strata. While a low PCV suggests the variable has little impact on the observed disparities across strata. This measure helps identify which variables are most influential in creating inequities or patterns in outcomes. Additionally, the PCV in Model 3 explains how much of the between-strata variance in Model 1 is explained by the additive and multiplicative effects (6). The PCV is expressed as a percentage by multiplying by 100, and is calculated using the following equation:

$$PCVs=\frac{\sigma_{\mathcal{u}(Model1)}^{2}-\sigma_{\mathcal{u}(Model2 or 3)}^{2}}{\sigma_{\mathcal{u}(Model1)}^{2}}$$

where $\sigma_{\mathcal{u}(Model1)}^{2}$ and $\sigma_{\mathcal{u}(Model2 or 3)}^{2}$​ represent the strata-level variance in the null model and the partially/main effect models, respectively. Consequently, 1−PCVs represents the percentage of the strata-level variance that cannot be explained by the additive effects and is therefore attributable to multiplicative effects (1,4).

**References**

1. Evans CR, Leckie G, Subramanian SV, Bell A, Merlo J. A tutorial for conducting intersectional multilevel analysis of individual heterogeneity and discriminatory accuracy (MAIHDA). SSM - Population Health [Internet]. junio de 2024 [citado 29 de julio de 2024];26:101664. Disponible en: https://linkinghub.elsevier.com/retrieve/pii/S235282732400065X

2. Evans CR, Williams DR, Onnela JP, Subramanian SV. A multilevel approach to modeling health inequalities at the intersection of multiple social identities. Social Science & Medicine [Internet]. abril de 2018 [citado 29 de julio de 2024];203:64-73. Disponible en: https://linkinghub.elsevier.com/retrieve/pii/S0277953617306664

3. Merlo J. Multilevel analysis of individual heterogeneity and discriminatory accuracy (MAIHDA) within an intersectional framework. Social Science & Medicine [Internet]. abril de 2018 [citado 29 de julio de 2024];203:74-80. Disponible en: https://linkinghub.elsevier.com/retrieve/pii/S0277953617307566

4. Keller L, Lüdtke O, Preckel F, Brunner M. Educational Inequalities at the Intersection of Multiple Social Categories: A n Introduction and Systematic Review of the Multilevel Analysis of Individual Heterogeneity and Discriminatory Accuracy (MAIHDA) Approach. Educ Psychol Rev [Internet]. marzo de 2023 [citado 29 de julio de 2024];35(1):31. Disponible en: https://link.springer.com/10.1007/s10648-023-09733-5

5. He JW, Terry AL, Lizotte D, Bauer G, Ryan BL. Understanding intersectional inequality in access to primary care providers using multilevel analysis of individual heterogeneity and discriminatory accuracy. Fiaschetti M, editor. PLoS ONE [Internet]. 19 de enero de 2024 [citado 29 de julio de 2024];19(1):e0296657. Disponible en: https://dx.plos.org/10.1371/journal.pone.0296657

6. Axelsson Fisk S, Mulinari S, Wemrell M, Leckie G, Perez Vicente R, Merlo J. Chronic Obstructive Pulmonary Disease in Sweden: An intersectional multilevel analysis of individual heterogeneity and discriminatory accuracy. SSM - Population Health [Internet]. abril de 2018 [citado 29 de julio de 2024];4:334-46. Disponible en: https://linkinghub.elsevier.com/retrieve/pii/S2352827317302033
