# Supplementary material for: Health inequities in functional limitation among Mexican older adults: An intersectional approach
Source: PLoS One. 2025 Aug 5;20(8):e0325211. doi: 10.1371/journal.pone.0325211 (PMC12324098; doi:10.1371/journal.pone.0325211)
Supplement: S2 File — (DOCX) [file pone.0325211.s002.docx]

**Supporting Information 2. Strata ranking.**

| Ranking | Strata ID | Age | Sex | At least one year of formal education | Social engagement | Economic status | Access to health services | n | Loss of functionality probability | 95%CI Upper limit | 95%CI Lower limit |
| --- | --- | --- | --- | --- | --- | --- | --- | --- | --- | --- | --- |
| 1 | 111111 | 50 - 59 | Male | With | With | Good | With | 403 | 8.31 | 6.42 | 10.7 |
| 2 | 121111 | 50 - 59 | Female | With | With | Good | With | 414 | 9.3 | 7.24 | 11.87 |
| 3 | 211111 | 60 - 69 | Male | With | With | Good | With | 312 | 9.31 | 7.17 | 12 |
| 4 | 111112 | 50 - 59 | Male | With | With | Good | Without | 122 | 9.58 | 7.15 | 12.72 |
| 5 | 121112 | 50 - 59 | Female | With | With | Good | Without | 87 | 10.06 | 7.49 | 13.38 |
| 6 | 211112 | 60 - 69 | Male | With | With | Good | Without | 84 | 10.21 | 7.58 | 13.62 |
| 7 | 121121 | 50 - 59 | Female | With | With | Fair | With | 528 | 10.91 | 8.69 | 13.62 |
| 8 | 112111 | 50 - 59 | Male | Without | With | Good | With | 11 | 11.38 | 8.19 | 15.6 |
| 9 | 112112 | 50 - 59 | Male | Without | With | Good | Without | 5 | 11.77 | 8.45 | 16.15 |
| 10 | 221111 | 60 - 69 | Female | With | With | Good | With | 234 | 11.97 | 9.25 | 15.36 |
| 11 | 221112 | 60 - 69 | Female | With | With | Good | Without | 43 | 12.18 | 9.03 | 16.25 |
| 12 | 121122 | 50 - 59 | Female | With | With | Fair | Without | 255 | 12.8 | 10 | 16.25 |
| 13 | 212111 | 60 - 69 | Male | Without | With | Good | With | 10 | 13.06 | 9.5 | 17.71 |
| 14 | 111121 | 50 - 59 | Male | With | With | Fair | With | 496 | 13.21 | 10.62 | 16.3 |
| 15 | 211122 | 60 - 69 | Male | With | With | Fair | Without | 247 | 13.68 | 10.71 | 17.33 |
| 16 | 111122 | 50 - 59 | Male | With | With | Fair | Without | 351 | 13.78 | 10.94 | 17.22 |
| 17 | 211121 | 60 - 69 | Male | With | With | Fair | With | 441 | 13.85 | 11.15 | 17.09 |
| 18 | 212112 | 60 - 69 | Male | Without | With | Good | Without | 14 | 13.95 | 10.13 | 18.89 |
| 19 | 111212 | 50 - 59 | Male | With | Without | Good | Without | 11 | 14.31 | 10.52 | 19.17 |
| 20 | 111211 | 50 - 59 | Male | With | Without | Good | With | 48 | 14.47 | 10.84 | 19.05 |
| 21 | 121211 | 50 - 59 | Female | With | Without | Good | With | 346 | 14.5 | 11.53 | 18.08 |
| 22 | 112121 | 50 - 59 | Male | Without | With | Fair | With | 20 | 14.51 | 10.67 | 19.43 |
| 23 | 222111 | 60 - 69 | Female | Without | With | Good | With | 9 | 14.8 | 10.83 | 19.9 |
| 24 | 211211 | 60 - 69 | Male | With | Without | Good | With | 186 | 14.84 | 11.52 | 18.91 |
| 25 | 221122 | 60 - 69 | Female | With | With | Fair | Without | 103 | 15.03 | 11.5 | 19.39 |
| 26 | 222112 | 60 - 69 | Female | Without | With | Good | Without | 11 | 15.28 | 11.16 | 20.57 |
| 27 | 121212 | 50 - 59 | Female | With | Without | Good | Without | 93 | 15.36 | 11.69 | 19.92 |
| 28 | 112122 | 50 - 59 | Male | Without | With | Fair | Without | 24 | 15.7 | 11.63 | 20.87 |
| 29 | 221121 | 60 - 69 | Female | With | With | Fair | With | 312 | 16.02 | 12.8 | 19.87 |
| 30 | 311112 | 70 - 79 | Male | With | With | Good | Without | 25 | 16.66 | 12.46 | 21.9 |
| 31 | 221211 | 60 - 69 | Female | With | Without | Good | With | 430 | 16.96 | 13.79 | 20.68 |
| 32 | 212121 | 60 - 69 | Male | Without | With | Fair | With | 33 | 16.99 | 12.71 | 22.33 |
| 33 | 211212 | 60 - 69 | Male | With | Without | Good | Without | 7 | 17.1 | 12.71 | 22.62 |
| 34 | 122122 | 50 - 59 | Female | Without | With | Fair | Without | 32 | 17.46 | 13.07 | 22.93 |
| 35 | 122121 | 50 - 59 | Female | Without | With | Fair | With | 20 | 17.79 | 13.26 | 23.45 |
| 36 | 311111 | 70 - 79 | Male | With | With | Good | With | 132 | 17.8 | 13.87 | 22.55 |
| 37 | 212122 | 60 - 69 | Male | Without | With | Fair | Without | 30 | 18.03 | 13.52 | 23.64 |
| 38 | 321111 | 70 - 79 | Female | With | With | Good | With | 78 | 18.36 | 14.09 | 23.57 |
| 39 | 121221 | 50 - 59 | Female | With | Without | Fair | With | 547 | 18.74 | 15.46 | 22.54 |
| 40 | 111222 | 50 - 59 | Male | With | Without | Fair | Without | 48 | 18.81 | 14.3 | 24.34 |
| 41 | 221212 | 60 - 69 | Female | With | Without | Good | Without | 51 | 19.37 | 14.81 | 24.92 |
| 42 | 121222 | 50 - 59 | Female | With | Without | Fair | Without | 250 | 19.44 | 15.55 | 24.02 |
| 43 | 111221 | 50 - 59 | Male | With | Without | Fair | With | 67 | 19.49 | 14.99 | 24.93 |
| 44 | 321112 | 70 - 79 | Female | With | With | Good | Without | 21 | 19.5 | 14.65 | 25.48 |
| 45 | 111132 | 50 - 59 | Male | With | With | Bad | Without | 36 | 19.5 | 14.63 | 25.52 |
| 46 | 222121 | 60 - 69 | Female | Without | With | Fair | With | 28 | 19.86 | 14.99 | 25.85 |
| 47 | 211131 | 60 - 69 | Male | With | With | Bad | With | 35 | 19.89 | 14.97 | 25.93 |
| 48 | 222122 | 60 - 69 | Female | Without | With | Fair | Without | 21 | 20.34 | 15.32 | 26.48 |
| 49 | 111131 | 50 - 59 | Male | With | With | Bad | With | 59 | 20.54 | 15.58 | 26.57 |
| 50 | 122212 | 50 - 59 | Female | Without | Without | Good | Without | 13 | 20.54 | 15.32 | 26.98 |
| 51 | 212211 | 60 - 69 | Male | Without | Without | Good | With | 5 | 20.98 | 15.64 | 27.54 |
| 52 | 122211 | 50 - 59 | Female | Without | Without | Good | With | 15 | 21.09 | 15.83 | 27.54 |
| 53 | 311121 | 70 - 79 | Male | With | With | Fair | With | 271 | 21.28 | 17.29 | 25.9 |
| 54 | 312111 | 70 - 79 | Male | Without | With | Good | With | 8 | 21.32 | 15.96 | 27.87 |
| 55 | 211222 | 60 - 69 | Male | With | Without | Fair | Without | 41 | 21.44 | 16.47 | 27.42 |
| 56 | 121131 | 50 - 59 | Female | With | With | Bad | With | 32 | 21.55 | 16.27 | 27.98 |
| 57 | 312112 | 70 - 79 | Male | Without | With | Good | Without | 15 | 22.23 | 16.66 | 29.01 |
| 58 | 121132 | 50 - 59 | Female | With | With | Bad | Without | 26 | 22.36 | 16.88 | 29.01 |
| 59 | 211132 | 60 - 69 | Male | With | With | Bad | Without | 37 | 22.4 | 16.95 | 28.99 |
| 60 | 222211 | 60 - 69 | Female | Without | Without | Good | With | 24 | 22.54 | 17.16 | 29.02 |
| 61 | 311122 | 70 - 79 | Male | With | With | Fair | Without | 119 | 22.71 | 17.96 | 28.27 |
| 62 | 221221 | 60 - 69 | Female | With | Without | Fair | With | 727 | 22.88 | 19.42 | 26.77 |
| 63 | 221222 | 60 - 69 | Female | With | Without | Fair | Without | 170 | 23.02 | 18.45 | 28.33 |
| 64 | 211221 | 60 - 69 | Male | With | Without | Fair | With | 246 | 23.62 | 19.23 | 28.67 |
| 65 | 322111 | 70 - 79 | Female | Without | With | Good | With | 5 | 24.47 | 18.46 | 31.68 |
| 66 | 221132 | 60 - 69 | Female | With | With | Bad | Without | 18 | 24.56 | 18.55 | 31.76 |
| 67 | 221131 | 60 - 69 | Female | With | With | Bad | With | 21 | 24.65 | 18.71 | 31.74 |
| 68 | 322112 | 70 - 79 | Female | Without | With | Good | Without | 6 | 25.2 | 18.98 | 32.63 |
| 69 | 321122 | 70 - 79 | Female | With | With | Fair | Without | 33 | 25.26 | 19.57 | 31.95 |
| 70 | 222212 | 60 - 69 | Female | Without | Without | Good | Without | 13 | 25.47 | 19.38 | 32.69 |
| 71 | 122221 | 50 - 59 | Female | Without | Without | Fair | With | 33 | 25.8 | 19.9 | 32.74 |
| 72 | 311212 | 70 - 79 | Male | With | Without | Good | Without | 19 | 26.88 | 20.81 | 33.97 |
| 73 | 122222 | 50 - 59 | Female | Without | Without | Fair | Without | 31 | 27.16 | 21.03 | 34.31 |
| 74 | 312122 | 70 - 79 | Male | Without | With | Fair | Without | 26 | 27.76 | 21.53 | 35 |
| 75 | 212132 | 60 - 69 | Male | Without | With | Bad | Without | 6 | 28.2 | 21.19 | 36.44 |
| 76 | 212222 | 60 - 69 | Male | Without | Without | Fair | Without | 9 | 28.27 | 21.72 | 35.9 |
| 77 | 312121 | 70 - 79 | Male | Without | With | Fair | With | 42 | 28.35 | 22.17 | 35.45 |
| 78 | 212221 | 60 - 69 | Male | Without | Without | Fair | With | 14 | 28.52 | 22.02 | 36.04 |
| 79 | 321121 | 70 - 79 | Female | With | With | Fair | With | 111 | 29.2 | 23.55 | 35.57 |
| 80 | 311221 | 70 - 79 | Male | With | Without | Fair | With | 468 | 29.24 | 24.89 | 34 |
| 81 | 311211 | 70 - 79 | Male | With | Without | Good | With | 269 | 29.27 | 24.37 | 34.71 |
| 82 | 111231 | 50 - 59 | Male | With | Without | Bad | With | 12 | 29.61 | 22.75 | 37.53 |
| 83 | 111232 | 50 - 59 | Male | With | Without | Bad | Without | 14 | 29.79 | 22.86 | 37.81 |
| 84 | 321211 | 70 - 79 | Female | With | Without | Good | With | 410 | 29.94 | 25.43 | 34.88 |
| 85 | 321212 | 70 - 79 | Female | With | Without | Good | Without | 49 | 30.02 | 23.81 | 37.06 |
| 86 | 322121 | 70 - 79 | Female | Without | With | Fair | With | 15 | 30.15 | 23.42 | 37.85 |
| 87 | 222221 | 60 - 69 | Female | Without | Without | Fair | With | 87 | 30.72 | 24.64 | 37.55 |
| 88 | 222132 | 60 - 69 | Female | Without | With | Bad | Without | 5 | 31.3 | 23.76 | 39.97 |
| 89 | 222222 | 60 - 69 | Female | Without | Without | Fair | Without | 43 | 31.34 | 24.81 | 38.71 |
| 90 | 121231 | 50 - 59 | Female | With | Without | Bad | With | 61 | 31.43 | 24.86 | 38.84 |
| 91 | 312211 | 70 - 79 | Male | Without | Without | Good | With | 25 | 31.65 | 24.84 | 39.33 |
| 92 | 322122 | 70 - 79 | Female | Without | With | Fair | Without | 10 | 31.77 | 24.74 | 39.74 |
| 93 | 121232 | 50 - 59 | Female | With | Without | Bad | Without | 39 | 32.64 | 25.67 | 40.47 |
| 94 | 211231 | 60 - 69 | Male | With | Without | Bad | With | 39 | 32.87 | 25.91 | 40.67 |
| 95 | 311132 | 70 - 79 | Male | With | With | Bad | Without | 10 | 32.93 | 25.47 | 41.36 |
| 96 | 311131 | 70 - 79 | Male | With | With | Bad | With | 16 | 33.39 | 26.03 | 41.67 |
| 97 | 411112 | 80 or more | Male | With | With | Good | Without | 5 | 33.42 | 25.89 | 41.91 |
| 98 | 321222 | 70 - 79 | Female | With | Without | Fair | Without | 145 | 34.09 | 28.21 | 40.49 |
| 99 | 411111 | 80 or more | Male | With | With | Good | With | 19 | 34.48 | 27.15 | 42.63 |
| 100 | 311222 | 70 - 79 | Male | With | Without | Fair | Without | 75 | 34.6 | 28.17 | 41.66 |
| 101 | 211232 | 60 - 69 | Male | With | Without | Bad | Without | 15 | 34.7 | 27.15 | 43.12 |
| 102 | 312212 | 70 - 79 | Male | Without | Without | Good | Without | 8 | 34.72 | 27.19 | 43.1 |
| 103 | 321131 | 70 - 79 | Female | With | With | Bad | With | 12 | 35.7 | 27.86 | 44.39 |
| 104 | 421111 | 80 or more | Female | With | With | Good | With | 11 | 36.05 | 28.32 | 44.59 |
| 105 | 322211 | 70 - 79 | Female | Without | Without | Good | With | 47 | 36.33 | 29.35 | 43.95 |
| 106 | 221232 | 60 - 69 | Female | With | Without | Bad | Without | 23 | 36.4 | 28.85 | 44.7 |
| 107 | 221231 | 60 - 69 | Female | With | Without | Bad | With | 72 | 36.45 | 29.48 | 44.04 |
| 108 | 322212 | 70 - 79 | Female | Without | Without | Good | Without | 28 | 37.21 | 29.77 | 45.32 |
| 109 | 321221 | 70 - 79 | Female | With | Without | Fair | With | 654 | 37.44 | 32.94 | 42.18 |
| 110 | 411121 | 80 or more | Male | With | With | Fair | With | 34 | 37.96 | 30.52 | 46.01 |
| 111 | 122231 | 50 - 59 | Female | Without | Without | Bad | With | 5 | 39.28 | 30.81 | 48.45 |
| 112 | 122232 | 50 - 59 | Female | Without | Without | Bad | Without | 5 | 39.81 | 31.31 | 48.98 |
| 113 | 312131 | 70 - 79 | Male | Without | With | Bad | With | 7 | 40.5 | 31.81 | 49.82 |
| 114 | 411122 | 80 or more | Male | With | With | Fair | Without | 22 | 40.64 | 32.7 | 49.1 |
| 115 | 312222 | 70 - 79 | Male | Without | Without | Fair | Without | 31 | 40.64 | 33.04 | 48.72 |
| 116 | 312221 | 70 - 79 | Male | Without | Without | Fair | With | 54 | 42.59 | 35.2 | 50.34 |
| 117 | 322221 | 70 - 79 | Female | Without | Without | Fair | With | 145 | 43.73 | 37.16 | 50.53 |
| 118 | 421121 | 80 or more | Female | With | With | Fair | With | 19 | 43.95 | 35.67 | 52.59 |
| 119 | 222232 | 60 - 69 | Female | Without | Without | Bad | Without | 9 | 44.22 | 35.48 | 53.33 |
| 120 | 421122 | 80 or more | Female | With | With | Fair | Without | 9 | 44.7 | 36.11 | 53.62 |
| 121 | 322222 | 70 - 79 | Female | Without | Without | Fair | Without | 66 | 44.78 | 37.43 | 52.36 |
| 122 | 311231 | 70 - 79 | Male | With | Without | Bad | With | 36 | 45.66 | 37.56 | 53.99 |
| 123 | 222231 | 60 - 69 | Female | Without | Without | Bad | With | 11 | 46.37 | 37.57 | 55.4 |
| 124 | 311232 | 70 - 79 | Male | With | Without | Bad | Without | 12 | 46.69 | 38.06 | 55.53 |
| 125 | 412122 | 80 or more | Male | Without | With | Fair | Without | 10 | 47.43 | 38.62 | 56.4 |
| 126 | 412121 | 80 or more | Male | Without | With | Fair | With | 7 | 47.65 | 38.81 | 56.64 |
| 127 | 411212 | 80 or more | Male | With | Without | Good | Without | 19 | 47.75 | 39.37 | 56.27 |
| 128 | 411211 | 80 or more | Male | With | Without | Good | With | 120 | 49.35 | 42.25 | 56.47 |
| 129 | 321231 | 70 - 79 | Female | With | Without | Bad | With | 50 | 50.95 | 42.87 | 58.99 |
| 130 | 321232 | 70 - 79 | Female | With | Without | Bad | Without | 16 | 51.65 | 42.94 | 60.25 |
| 131 | 421212 | 80 or more | Female | With | Without | Good | Without | 32 | 52.17 | 43.91 | 60.31 |
| 132 | 411221 | 80 or more | Male | With | Without | Fair | With | 200 | 52.68 | 46.15 | 59.13 |
| 133 | 411131 | 80 or more | Male | With | With | Bad | With | 5 | 53.6 | 44.12 | 62.84 |
| 134 | 411222 | 80 or more | Male | With | Without | Fair | Without | 46 | 54.08 | 46.11 | 61.84 |
| 135 | 412211 | 80 or more | Male | Without | Without | Good | With | 23 | 54.79 | 46.22 | 63.08 |
| 136 | 312231 | 70 - 79 | Male | Without | Without | Bad | With | 5 | 55.05 | 45.9 | 63.88 |
| 137 | 412212 | 80 or more | Male | Without | Without | Good | Without | 9 | 55.15 | 46.2 | 63.78 |
| 138 | 312232 | 70 - 79 | Male | Without | Without | Bad | Without | 10 | 55.72 | 46.57 | 64.5 |
| 139 | 421211 | 80 or more | Female | With | Without | Good | With | 199 | 56.11 | 49.55 | 62.45 |
| 140 | 422212 | 80 or more | Female | Without | Without | Good | Without | 25 | 57.41 | 48.87 | 65.53 |
| 141 | 322232 | 70 - 79 | Female | Without | Without | Bad | Without | 9 | 58.88 | 49.9 | 67.31 |
| 142 | 322231 | 70 - 79 | Female | Without | Without | Bad | With | 17 | 59.52 | 50.74 | 67.73 |
| 143 | 422211 | 80 or more | Female | Without | Without | Good | With | 42 | 59.73 | 51.66 | 67.31 |
| 144 | 421222 | 80 or more | Female | With | Without | Fair | Without | 65 | 59.84 | 52.23 | 67.01 |
| 145 | 412221 | 80 or more | Male | Without | Without | Fair | With | 52 | 60.84 | 52.96 | 68.18 |
| 146 | 421221 | 80 or more | Female | With | Without | Fair | With | 225 | 61.37 | 55.13 | 67.25 |
| 147 | 412222 | 80 or more | Male | Without | Without | Fair | Without | 29 | 63.13 | 55.02 | 70.57 |
| 148 | 422222 | 80 or more | Female | Without | Without | Fair | Without | 40 | 64.65 | 56.9 | 71.71 |
| 149 | 422221 | 80 or more | Female | Without | Without | Fair | With | 97 | 66.07 | 59.03 | 72.46 |
| 150 | 411231 | 80 or more | Male | With | Without | Bad | With | 16 | 67.43 | 59.14 | 74.77 |
| 151 | 411232 | 80 or more | Male | With | Without | Bad | Without | 8 | 68.1 | 59.61 | 75.54 |
| 152 | 421232 | 80 or more | Female | With | Without | Bad | Without | 12 | 71.14 | 63.08 | 78.05 |
| 153 | 421231 | 80 or more | Female | With | Without | Bad | With | 13 | 72.54 | 64.84 | 79.1 |
| 154 | 412231 | 80 or more | Male | Without | Without | Bad | With | 11 | 73.82 | 65.95 | 80.42 |
| 155 | 412232 | 80 or more | Male | Without | Without | Bad | Without | 9 | 74.03 | 66.12 | 80.63 |
| 156 | 422231 | 80 or more | Female | Without | Without | Bad | With | 14 | 77.45 | 70.32 | 83.27 |
| 157 | 422232 | 80 or more | Female | Without | Without | Bad | Without | 11 | 77.95 | 70.85 | 83.71 |
